# Retrospect of the Medical Sciences

**Published:** 1843-11-11

**Authors:** 


GANGRENE OF THE LUNG, INTESTINAL OBSTRUCTIONS, ETC.

At the late meeting of the British Association for the Advancement of Science, held at Cork, Dr. Popham related three cases of gangrene of the lung that had fallen under his care. Two of them had proved fatal, but the last was cured by administering, internally, the chloride of lime. In this case the patient appeared to be sinking, when the chloride, with tincture of opium, was administered. In a short time favourable symptoms came on, and the patient quickly recovered.

Dr. Oliffe read a paper on "Intestinal Obstructions," pointing out the value of the operation for artificial anus and obstinate constipation, as proposed by Amussat, and giving some account of the eight cases in which Amussat had operated, and detailing one of his own, all of them having been successfully treated.

Dr. O'Connor gave the particulars of a case in which a boy, twelve years of age, had lost all the hair of his head after a "frightful dream," the scalp, after seven years, still remaining bald. The only hair on his body was a few straggling woolly hairs, in the place of whiskers.—*London Lancet*, Sept 16th, 1843.

### RETROSPECT OF THE MEDICAL SCIENCES.

### STATISTICS ON THE MORTALITY AFTER AMPUTATION.

In the service of M. Jobert at the Hôpital St. Louis, 31 capital amputations have been performed within the last two years and a half, with only 17 deaths. The amputations of the fingers and toes are not included, but although the number was considerable during the above period, no instance of death after them occurred.

| | Amputations. | Deaths. |
|---|---|---|
| Thigh, | 11 | 5 |
| Leg, | 6 | 2 |
| Of Foot, (partial amp.) | 4 | 0 |
| Arm, | 3 | 0 |
| Forearm, | 3 | 0 |
| Disarticulation of Shoulder, | 3 | 0 |
| Resection of Elbow Joint, | 1 | 0 |
| Total, | 31 | 7 |

M. Jobert has performed disarticulation of the shoulder joint nine times, and only two cases have terminated fatally.

All M. Jobert's amputations are flap-operations. In the supra-malleolar amputation, he takes his flap from the posterior soft parts, which he applies to a small flap formed by dissecting up the skin, and which latter extends about two centimetres and a half beyond the point of section of the bones. The cicatrix is thus formed on the anterior portion of the leg, and the point of support is a thick cushion of flesh, constituted by the posterior flap.—*Gazette des Hôpitaux.*

---

### URINE IN MORBUS BRIGHTII.

BY DR. F. SIMON, OF BERLIN.

About a year and a half ago my attention was called to the peculiar sediment which separates from the albumen of the urine in certain cases of albuminuria, particularly in cases where Bright's degenerescence of the kidneys happens to be present, and which, on being examined with the microscope, seems of a peculiar kind, and entirely different from the ordinary sediments. This appears to the naked eye as a slightly mucous deposit, not, however, to be confounded with the sediment from the vesical mucus; if the urine be carefully poured off, and the sediment be brought to the object-glass under the microscope, the following various forms are observed:—

1. Mucus-corpuscles of the ordinary size, partly granulated, with grains readily recognised.

2. Portions of epithelium, from the vesical mucous membrane.

3. Blood-corpuscles.

4. Round, dark globules, apparently filled with a granular substance of a diameter between 0.00060 and 0.00090 parts of an inch. These globules bore the greatest resemblance to those designated by Gluge inflammation-globules.

5. Vesicles, or vesicle-like arrangement of an amorphous matter, like coagulated albumen. These vesicles, for the most part, had real coverings, and were cylindrical, but in some the coverings seemed to be wanting, and it was then only perceived that an amorphous, slightly granulated matter was connected together in a cylindrical form. The cylinders were in part entirely filled, in part they were empty, and contained only a small quantity of a granular substance. The filled cylinders contained sometimes a light, sometimes a dark, granular substance, in which cells and globules, like mucus-corpuscles, appeared to exist, of the real presence of which, however, I was not able always to satisfy myself with perfect certainty. The breadth of the cylinder differed somewhat; the broadest I estimated at 0.00110 parts of an inch, the smaller at 0.00060. My attention being once directed to this phenomenon, I examined the urine attentively in cases of albuminuria, where pains in the region of the kidney indicated disease of this organ; and also in several cases where a degenerescence was admitted to exist, I observed these vesicles and globules. There is no doubt that these peculiar forms had their origin in the kidneys, and they are probably the morbidly changed epithelial linings of Bellini's urinary canals. Whether they occur only in cases of Bright's disease, or in other cases also, requires to be ascertained by more extended experience; according to my observations, they always appear at the same time with a certain amount of albumen in the urine, but the blood-corpuscles are not always present. In one case, where ascites and anasarca had already occurred, and the urine was secreted in only small quantity, I twice made a chemical examination of the urine: the first time, the quantity of urine voided in twenty-four hours amounted to twelve ounces; it was of a dark-brown color; contained no blood, but so much albumen that, after boiling in a test-glass, the matter might be inverted without the coagulated albumen running out; the urine had an acid reaction, contained in the white mucous sediment much of the above-mentioned vesicles and globules, and had a specific gravity of 1.014. After some days the quantity of the secreted urine was less, its specific gravity greater, and it still continued to have an acid reaction. In both cases the uric acid was in a tolerably normal quantity, but the quantity of urea was diminished; some days later, with the diminished quantity of albumen, yellow-colored uric acid presented itself, secreted spontaneously in large rhombic plates. I think that the occurrence of this peculiar form in albuminous urine, in cases of Bright's renal degenerescence, is of sufficient importance to invite the attention of the physician.—*Prov. Med. Journ.*, Sept. 9, 1843.

---

### INFLUENCE OF THE SEASONS.

The results to which the foregoing facts and reasonings lead, may be briefly stated as follows:—

1. The amount of sickness in the central districts of London, during the year 1842, varied directly as the temperature, being a maximum in August, the hottest month of the year, and a minimum in January, the coldest month.

2. The diseases which determined the order of sickness, were febrile and catarrhal affections, the contagious exanthemata, and the disorders of the digestive organs; to which may be added the mixed group, consisting of gout, scrofula, &c.

3. The diseases of the organs of respiration followed the inverse order of those already mentioned, and were inversely as the temperature, being most numerous in the colder, and fewest in the hotter months.

4. The temperature did not appear to exercise a marked influence on the other classes of disease: with the exception, perhaps, of those which form a measure of the activity of the sexual passion, which were in excess during the hottest months of the year—a fact which corresponds with, and corroborates, our experience of the influence of the seasons on crimes against the person, &c.

5. The hygrometric state of the air appeared to have little effect on disease; and, if it produced any effect, it was on the diseases of the organs of respiration, which were in excess during the months in which the quantity of moisture in the air was the greatest; but these were also the coldest months.

6. The mortality for the metropolis during the year 1842, was greatest in the first quarter, and least in the second, and was inversely as the sickness, except that the mortality of the third quarter exceeded that of the fourth.

7. The diseases which chiefly influenced the order of the quarters in respect of mortality, were those of the chest; to which may be added, as following the same order, the decay of nature in the aged. It is well known that the most common cause of death, in the aged, is an affection of the lungs, called "bronchitis senilis."

8. The order of the seasons in respect of sickness and mortality differs year by year, and does not admit of being reduced to any precise rule.

9. As a general rule, but one admitting of many exceptions, it may be stated, that the amonnt of sickness tends to vary directly, and the amount of mortality inversely as the temperature.

These results must be received with some reserve, as they are founded on a comparatively small number of facts; but they are, probably, not very far from the truth. At any rate they may prove suggestive of future inquiries, founded upon a broader basis. At present the materials for a comprehensive theory of the influence of the seasons and weather upon sickness and mortality are wanting, and are not likely to be supplied till the example set by one or two public hospitals and dispensaries shall have provoked imitation. In the meantime, the present attempt, if it accomplish no other purpose, may serve as an example of the mode by which such inquiries must be conducted.

In the course of this inquiry it is scarcely possible that some hypothesis should not have suggested itself as the most likely to prove true; and it may not be amiss to bring this attempt to a conclusion, by stating in a few words that which I have been led to form.

The causes of sickness are twofold, consisting of atmospheric changes which may be submitted to measurement, and of certain more subtle changes in the composition of the air, which, at present, can neither be analysed nor estimated. To the former class belong the temperature, moisture and pressure, of the air; to the latter those emanations from the earth, or from human beings themselves, which give rise to the majority of epidemic, endemic, and contagious diseases. As the number of cases of sickness produced by these latter causes, is generally considerable, the influence of the pressure, temperature, and hygrometric state of the air, will not be observed in those years in which these causes are in operation; but in the absence of epidemics, the temperature will be found to be the most influential cause of sickness. When the temperature of the summer is high, there will be such an amount of sickness in the summer months as to cause a large return of sickness for the entire year; so, on the other hand, a severe winter will swell the total sickness of the year, by producing a great excess of affections of the organs of respiration. A summer or winter of unusual length, beginning early and ending late, will also cause an increase of sickness on the entire year; but the nature of the sickness will be different as the temperature is higher or lower than usual. The order of the seasons in respect of sickness will also be mainly determined by the degree in which the temperature of these seasons exceeds, or falls short of, the average temperature.

The mortality, in like manner, in non-epidemic years will be chiefly dependent upon the temperature, varying in the several seasons inversely as the temperature, except in those years in which the summer is unusually warm, when the mortality of the summer may even exceed that of the winter season. In other instances, the mortality of the summer months will rank next to that of the winter or autumn.—*Dr. Guy, in the Quarterly Journal of the Statistical Society.*

---

### URINE IN DISEASES OF THE SPINAL CORD.

BY DR. SIMON, OF BERLIN.

It is well known that in affections of the spinal cord, as also of the brain, the urine very frequently comes to have an ammoniacal reaction, and, on adding a free acid, is found to contain a considerable quantity of carbonate of ammonia by its effervescence. It was supposed for a long time that the carbonate of ammonia was formed at the expense of the urea, by means of the latter taking the elements of two atoms of water. However, as other nitrogenised substances also present themselves in the urine, the formation of the carbonate of ammonia may possibly proceed from them; accordingly, I deemed it not uninteresting to ascertain by direct experiment whether the urea has any share in the occurrences of this change or not. With this view I examined, at different times, the urine of a person who was suffering much from spinal disease. At first, with the carbonate of ammonia I detected urea also, in much smaller quantity, however, than in normal urine; afterwards, as the morbid symptoms continually became worse, and the urine was found to be ammoniacal even when secreted, the quantity of the urea became more and more diminished, and on examining the urine once more, which now exhaled a most offensive odor, a short time before the patient's death, I found the quantity of urea so much diminished, that on adding nitrate of potash, it was not till after several hours crystals of nitrate of urea separated from the alcoholic extract of the urinary residue: hence it follows that the formation of the carbonate of ammonia goes on at the expense of the urea. It seems to me important to observe, that in disease of the spinal cord or brain, when, in consequence of the formation of carbonate of ammonia, the earthy phosphates are separated, among these latter the triple phosphate of magnesia predominates in the generality of cases; I previously remarked, that in the resolution of pleuro-pneumonia I have on some occasions observed sediments merely of phosphate of ammonia and magnesia. In disease of the mucous membrane of the bladder, on the contrary, when the earthy phosphates are separated from the urine, the phosphate of lime prevails. The qualities of the separated earthy phosphates are oftentimes much too great to admit of being accounted for by the precipitation of the ordinary contents of the urine. Dr. Prout remarks, that here a connection takes place between the phosphoric contents of the cerebral fat on the one hand, and the phosphate of lime, and of the mucous membrane and of the mucus on the other hand; these are points which certainly merit attention.—*Provincial Medical Journal, from Beiträge Zur Physiologischen und Pathologichen, Chemie, und Mikroskopie.*

